# Auditory Conflict Resolution Correlates with Medial–Lateral Frontal Theta/Alpha Phase Synchrony

**DOI:** 10.1371/journal.pone.0110989

**Published:** 2014-10-24

**Authors:** Samantha Huang, Stephanie Rossi, Matti Hämäläinen, Jyrki Ahveninen

**Affiliations:** 1 Harvard Medical School, Athinoula A. Martinos Center for Biomedical Imaging, Department of Radiology, Massachusetts General Hospital, Charlestown, Massachusetts, United States of America; 2 Harvard–MIT Division of Health Sciences and Technology, Cambridge, Massachusetts, United States of America; University of British Columbia, Canada

## Abstract

When multiple persons speak simultaneously, it may be difficult for the listener to direct attention to correct sound objects among conflicting ones. This could occur, for example, in an emergency situation in which one hears conflicting instructions and the loudest, instead of the wisest, voice prevails. Here, we used cortically-constrained oscillatory MEG/EEG estimates to examine how different brain regions, including caudal anterior cingulate (cACC) and dorsolateral prefrontal cortices (DLPFC), work together to resolve these kinds of auditory conflicts. During an auditory flanker interference task, subjects were presented with sound patterns consisting of three different voices, from three different directions (45° left, straight ahead, 45° right), sounding out either the letters “A” or “O”. They were asked to discriminate which sound was presented centrally and ignore the flanking distracters that were phonetically either congruent (50%) or incongruent (50%) with the target. Our cortical MEG/EEG oscillatory estimates demonstrated a direct relationship between performance and brain activity, showing that efficient conflict resolution, as measured with reduced conflict-induced RT lags, is predicted by theta/alpha phase coupling between cACC and right lateral frontal cortex regions intersecting the right frontal eye fields (FEF) and DLPFC, as well as by increased pre-stimulus gamma (60–110 Hz) power in the left inferior fontal cortex. Notably, cACC connectivity patterns that correlated with behavioral conflict-resolution measures were found during both the pre-stimulus and the pre-response periods. Our data provide evidence that, instead of being only transiently activated upon conflict detection, cACC is involved in sustained engagement of attentional resources required for effective sound object selection performance.

## Introduction

Speech perception in everyday acoustic environments is a considerable computational challenge. When several persons speak at the same time, the spectrotemporal overlap of competing signals may hinder the segregation of the competing sound streams to form distinct perceptual objects (*i.e.,* object formation) [1]. Even if the relevant and irrelevant sounds can be perceptually grouped into distinct objects, similarity of these objects can make it difficult for the listener to direct attention to correct object in the auditory scene (*i.e*., object selection) [1]. This could happen, for example, when one hears conflicting pieces of advice in an emergency situation, and the inherently most salient instead of most relevant object tends to prevail. While the top-down modulations of auditory neurons supporting perceptual object formation (for a review, see [2]) under the control of higher-order frontoparietal areas (for reviews, see [3], [4]) have been documented, a paucity of information exists on how the higher-order brain regions work together to support selection of relevant sound objects amongst competing or conflicting sound objects.

The question about how different brain regions work together to select relevant objects while ignoring conflicting stimuli in a multitalker environment falls within the cognitive realm of conflict processing. In the visual domain, conflict processing has been intensively studied using the classic Flanker task [5], in which flanking distractor letters or arrows interfere with judgments about central target letters or target arrow direction, respectively. Much like in a multitalker setting where more salient voices can be assumed to complicate speech-object selection in the auditory domain [1], the more salient/numerous flankers significantly interfere with the selection of the relevant visual object and the motor responses associated with it. In addition to the Flanker task, other widely used conflict tasks include the Stroop color-word task [6], requiring the subject to identify the color of the letters instead of reading the word, and the Simon spatial compatibility task where a conflict arises when subjects need to respond with the hand opposite to the side of screen where a visual target appears [7]. A large body of evidence from human neuroimaging studies, using different tasks (although mainly visual) typically report activations in anterior cingulate cortex (ACC), lateral prefrontal cortices (PFC) (including dorsolateral PFC, DLPFC, and inferior frontal cortex, IFC), anterior insula (AI), and parietal cortices [8]–[14]. However, given the methodological limitations, such as the poor temporal resolution of the prevailing neuroimaging technique of fMRI, it is not completely clear how these regions work together as a functional network during conflict processing.

In order to disentangle specific processes and mechanisms that underlie attentional control during conflict resolution, it is necessary to use imaging tools with high temporal resolution, such as magnetoencephalography (MEG) and EEG that provide a direct measure of post-synaptic neuronal processes. Findings from event-related potential (ERP) studies using various conflict tasks suggest that the components N200 (also known as N2) [15] and N450 [16] are related to stimulus-level conflict, while the error-related negativity (ERN) component [15] reflects response-level conflict (for a review, see [17]). A limitation of such conventional ERP analyses are that task-modulated non-phase-locked “induced” dynamics of EEG data are not-present trial-averaged responses, and that they do not allow inferences of interregional dynamics during conflict processing [18]. However, accumulating evidence suggests that functional coupling across distant brain areas during attention and cognitive control can be explicitly quantified based on interregional phase locking of neuronal oscillations [19], [20], occurring at distinct frequency ranges. It appears that low-frequency (<30 Hz) oscillations at the theta (4–7 Hz), alpha (7–15 Hz), and beta (15–30 Hz) ranges are particularly relevant for longer-range coupling associated with top-down modulation of neurons [21]–[23]. Evidence from human studies suggests that such coupling may be enhanced in task-relevant networks during auditory attention [24], as well as visually-delivered working memory [25] and conflict-processing tasks [26], [27]. It has been also noninvasively shown that, during auditory orienting, interregional interactions at the gamma band are modulated by the phase of theta in humans [28]. As suggested by extracellular measurements in cats [29] and human MEG studies [30], inter-regional phase locking may also increase at the beta band during attention and/or cognitive control tasks.

Previous auditory fMRI studies suggest that ACC is essential for selective attention to task-relevant sound features [31], [32] and auditory feedback monitoring [33]. It is specifically activated when the subject is asked to pay attention selectively to one of two dichotically presented phonemes and ignore a simultaneously presented competing stimulus [34]. However, only a small number of auditory behavioral [35], [36] and neuroimaging [32], [34] studies on perceptual conflicts have been conducted in the auditory domain, especially at the early stage of object identification and selection, that is, the stimulus conflict. Here, we therefore tested the hypothesis that ACC may also support selective listening in complex auditory settings such as multitalker environments. To achieve this goal, we used non-invasive estimates of neuronal oscillations [24], [37], [38] to investigate the ACC and DLPFC oscillatory phase locking patterns predicting fast and efficient conflict processing using a modified auditory flanker interference task, in which participants were required to identify a spoken “A” or “O” from acoustically similar or distinct flankers. Source localization of neuronal oscillations was facilitated by using anatomical MRI constraints to limit the potential solutions to the cortical gray matter [39], [40], where non-invasively measurable MEG and EEG activities are generated [41], and by combining the complementary information provided by simultaneously measured MEG and EEG [42]. A priori regions of interest (ROIs) including the caudal ACC (cACC) and DLPFC in each hemisphere were first defined based on the Desikan-Killiany atlas [43]. The cACC seed roughly corresponded to the anterior mid-cingulate cortex area that has also been termed “dorsal ACC” in previous conflict processing studies [44]. Seed-based oscillatory phase locking estimates between these ROIs and all cortical locations were then calculated using the debiased weighted phase lag index (wPLI), to test the specific hypothesis that conflict processing is associated with increased cortico-cortical low-frequency phase locking between ACC and lateral prefrontal regions.

## Materials and Methods

### Participants

The study protocol was approved by the Partners Human Research Committee, the Institutional Review Board (IRB) of the Massachusetts General Hospital. Potential subjects were first screened with a phone interview to ensure that they had normal hearing and had not been exposed regularly to environments with excessively loud noise. Twelve right-handed college-level educated adults with normal hearing and no neurological disorders, psychiatric conditions, or learning disabilities, gave written informed consent prior to testing.

### Task and stimuli

**Figure 1:**
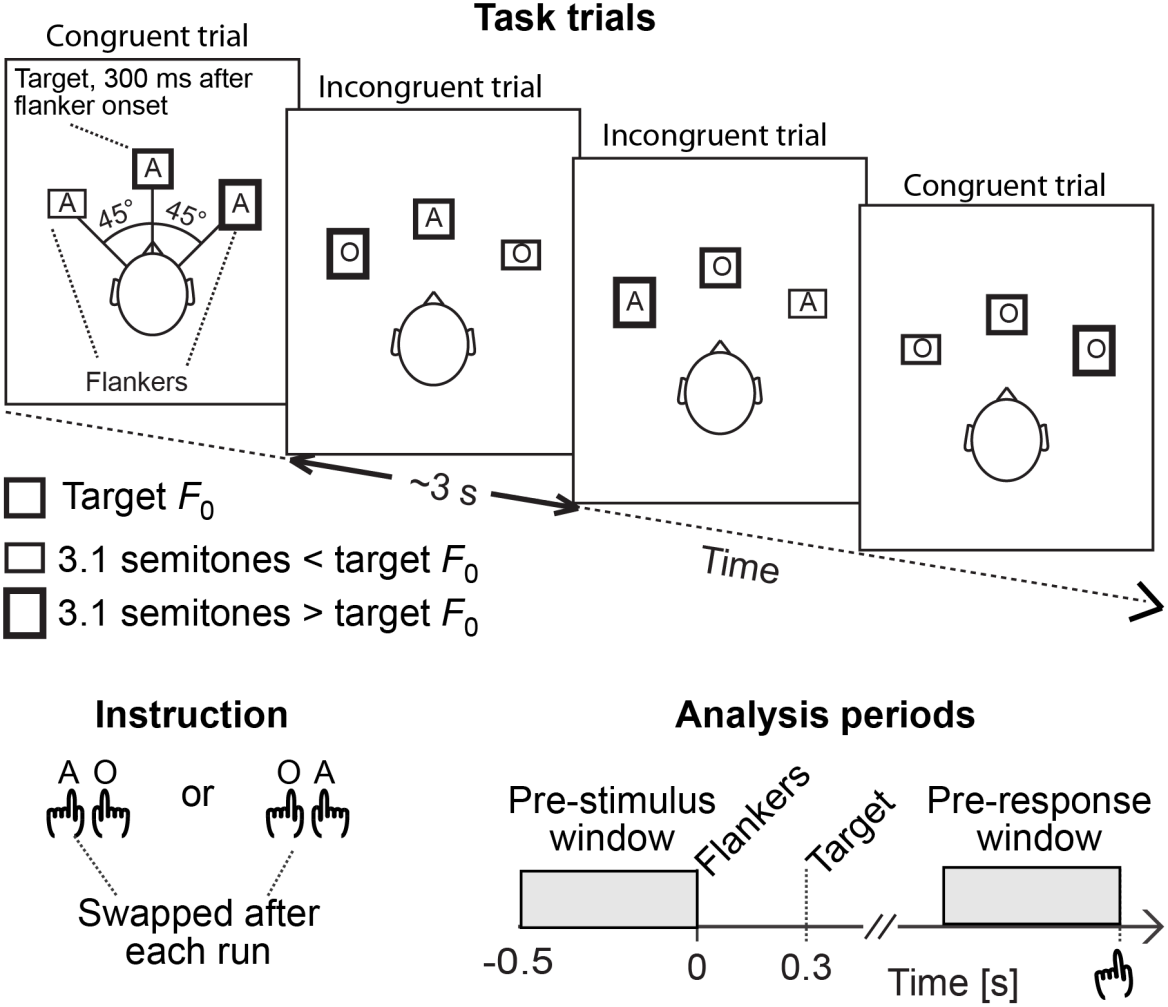
Task design and oscillatory analysis periods. *Task trials*: Subjects were instructed to pay attention to the target sounds and ignore flanking distractor sounds, which were phonetically either congruent (50%) or conflicting (50%) with the target. The flankers arrived 45° to the right and left of the target, at a 3.1 semitones lower or higher pitch than the target. *Instruction*: In a counterbalanced order across runs/subjects, subjects were asked to press a button either with the left index finger to “A” and with the right index finger to “O”, or vice versa. *Analysis periods*: To determine oscillatory power and phase locking patterns that predict efficient conflict processing, the analyses were concentrated on the time periods that preceded each stimulus trial (“Pre-stimulus window”) and each behavioral response (“Pre-response window”).

During MEG/EEG acquisitions, subjects (*N* = 12, age 20–33 years, 6 females) were presented with an auditory spatial-phonetic flanker interference task modified based on previous psychoacoustic studies [35] (**Figure 1**). Each sound trial, presented at a randomly varying inter-trial onset interval of 2.95–3.05 s, included two 400 ms “flankers” simulated horizontally from 45° to the right and left using generic head-related transfer functions [45], and a 400 ms target sound coming from straight ahead that started 300 ms after the flankers. The delay was introduced to the target, as very few subjects were able to discriminate the target from simultaneous flankers. The setting was thus analogous to certain previous visuospatial flanker studies [46], [47], with the additional delay being also justified because of the serial nature of phonetic sound-object processing [48]. The flankers and targets consisted of a voice sounding out the American English letters “A” or “O”, obtained from the Psychology Experiment Building Language (PEBL) Sound Archive version 0.1. The pitch of the flankers was digitally manipulated to be 3.1 semitones lower or higher than the target, such that the listener always heard three sounds coming from three different directions at three different pitches. Equiprobably, the subject heard either a congruent set of voices (“A_Left_-*A_Middle_*-A**_Right_**” or “O_Left_-*O_Middle_*-O_Right_”) or an incongruent/conflicting set of voices (“A_Left_-*O_Middle_*-A_Right_” or “O_Left_-*A_Middle_*-O_Right_”). In all trials, subjects were instructed to ignore the flanking distractors and pay attention only to the middle target by pressing a button with the index finger of one hand if the target was “A” and with the index finger of the opposite hand if the target was “O”. The hand/target letter order was switched after each of two 16-minute runs (324 trials per run), in an counterbalanced order across the subjects. Subjects’ responses were recorded together with the MEG/EEG raw data and reaction time (RT) was measured from the moment of the target appearance to the moment of button pressing. Two subjects were excluded from the final MEG/EEG analyses because they could not perform the tasks.

### Data acquisition

We recorded 306-channel MEG (Elekta-Neuromag, Helsinki, Finland) and 74-channel EEG data simultaneously in a magnetically shielded room (sampling rate 1 kHz, passband 0.03–330 Hz). The average reference was utilized for all analyses of EEG data. The position of the head relative to the MEG sensor array was monitored continuously using four head position indicator (HPI) coils attached to the scalp. Electrooculogram (EOG) was also recorded to monitor eye artifacts. T1-weighted structural MRIs were obtained for combining anatomical and functional data using a multi-echo MPRAGE pulse sequence (TR = 2510 ms; 4 echoes with TEs = 1.64 ms, 3.5 ms, 5.36 ms, 7.22 ms; 176 sagittal slices with 1×1×1 mm^3^ voxels, 256×256 mm^2^ matrix; flip angle = 7°). Multi-echo FLASH data were obtained with 5° and 30° flip angles (TR = 20 ms, TE = 1.89+2n ms [n = 0–7], 256×256 mm^2^ matrix, 1.33-mm slice thickness) for reconstruction of head boundary-element models (BEM).

### Data analysis

Neuronal bases of auditory conflict processing were studied using a cortically constrained MEG/EEG MNE approach [24], [38]. The MNE method reconstructs the intracranial sources of activity by identifying the distribution of dipoles with minimum total power that accounts for the magnetic field distribution recorded over the scalp. The cortically constrained approach takes into account the shape of the cortex and thus provides a better spatial resolution than an MNE only confined to the intracranial volume [39], [40]. Our overall workflow consists of (i) preprocessing of the MEG/EEG data (removal of artifacts and estimation of noise covariance matrix), (ii) preprocessing of the anatomical MRI data (reconstruction of the cortical surface to set up individual MEG/EEG source space, and the reconstruction of the scalp, outer skull, and inner skull surfaces), (iii) alignment of the MEG and MRI coordinate frames and computing forward solutions, and (iv) computing the MNE inverse operator for source estimates.

External MEG noise was suppressed and subject movements, estimated continuously at 200-ms intervals, were compensated for using the signal-space separation method [49] (Maxfilter, Elekta-Neuromag, Helsinki, Finland). The MEG/EEG data were downsampled (333 samples/s, passband 0.5–110 Hz). Epochs coinciding with over 150 µV EOG, 100 µV EEG, 2 pT/cm MEG gradiometer, or 4 pT MEG magnetometer peak-to-peak signals were excluded from further analyses.

For building the cortically constrained source space, we used FreeSurfer 5.3 [50] (http://surfer.nmr.mgh.harvard.edu/) to determine the shapes of the surfaces separating the scalp, skull, and brain compartments, and to build the triangular cortical surface mesh from T1-weighted anatomical 3D-volume MRI data by exploiting the gray-white matter boundary generated by the software. The FreeSurfer software was used due to the following advantages [50]–[52]: first, in addition to folded surfaces, it also computes inflated and flattened representations of the cortex, and can thus expose the parts of the cortex embedded in the sulci. Further, it provides an automated parcellation of the cortex that can be used in inquiring source waveforms in specific ROI. Finally, an additional benefit of the surface-based analysis is that cortical surfaces can be aligned across individuals for the computation of group statistics using a morphing procedure in a spherical coordinate system [50] and several recent studies have shown that the method has the highest stability in gray matter volume estimate compared to two other volume-based methods [53], and better alignments of cortical landmarks than volume-based registrations [54]. To achieve sufficient anatomical detail for the visualization of the folded cortical mantle, the triangular tessellations of each hemisphere contain roughly 100,000 potential vertices, spaced at ∼1 mm. For inverse computations, the dense triangulations were decimated to ∼1,000 vertices per hemisphere for computational efficiency.

To calculate MRI-guided depth-weighted ℓ_2_ MNE [55], the information from structural segmentation of the individual MRIs and the MEG sensor and EEG electrode locations were used to compute the forward solutions for all putative source locations in the cortex using a three-compartment BEM [55].

The covariance matrix and the forward solution computed in the previous procedures were used to obtain a distributed cortically constrained minimum-norm inverse operator that relates the sensor measurements to dipole current estimates in the source space. The individual forward solutions for current dipoles placed at these vertices comprised the columns of the gain matrix (**A**). A noise covariance matrix (**C**) was estimated from the raw MEG/EEG data during a 20–200-ms pre-stimulus baseline. These two matrices, along with the source covariance matrix (**R**), were used to calculate the MNE inverse operator **W** = **RA** ^T^ (**ARA** ^T^ + **C**)^−1^, which is applied to the sensor-level data to yield the source time courses.

To investigate seed-based cortico-cortical phase locking patterns, the entire MEG/EEG raw data time series at each time point were multiplied by the inverse operator **W** and noise normalized to yield the estimated source activity as a function of time across the entire cortex [40]. In addition, the frontal seed regions, including cACC and DLPFC, were selected from each hemisphere using the Freesurfer anatomical atlas [43]. The DLPFC was, specifically, defined based on the “rostral middlefrontal cortex” label [43], which encompasses the junction of Brodmann’s areas 9, 10, and 46 [56]. An average raw data time course was then calculated within these seed regions, with the waveform signs of sources aligned on the basis of surface-normal orientations to avoid phase cancellations.

Accepted MEG/EEG trial epochs were analyzed using the MNE and MNE-Python [57], and FieldTrip tools (http://www.ru.nl/fcdonders/fieldtrip). To investigate the phase locking between the cACC and DLPFC seed regions and all cortical locations, a FFT with Hanning window was applied on (**a**) the raw data epochs in each vertex of the right and left hemisphere and (**b**) on the corresponding trials of the pooled raw time courses of the right and left seed regions, separately for the 500-ms periods preceding each sound triplet and each behavioral response. Our presumption was that long-range inter-regional phase locking patterns would occur in theta and alpha, and lower beta ranges. Therefore, the FFT was applied at 2-Hz intervals at 6–20 Hz at the 500-ms windows and a Hanning taper. The 500-ms window length was selected on the basis of the RT data, such that the analysis window would not overlap with the target sound onset. We then calculated the phase locking between the seeds and each vertex the left and right hemisphere using the debiased wPLI. In addition to controlling for the sample-size bias, the debiased wPLI provides an index of nonzero phase lags across the regions/locations of interest that helps avoid spurious inflation of synchronization indices due to EEG and MEG point spread and crosstalk [58]. The resulting seed-based debiased wPLI estimates, mapped across all cortical locations in each subject, were then normalized to the Freesurfer standard brain representation for surface-based group statistical analyses [59].

In addition to the main analysis concentrating on the seed-based phase locking, we also estimated the power of neuronal oscillations across the available band during 500-ms pre-stimulus and pre-response periods, using a FFT with a Hanning taper at 2-Hz intervals between 4 and 30 Hz and an adaptive time-window of 3 cycles. At 30–110 Hz, 3-Hz frequency smoothing was used, resulting in three orthogonal Slepian tapers being applied to the 500-ms time window [60]. Analogous to [38], the available frequency band was divided to consecutive one-octave wide sub ranges, which corresponded to theta (4–7.5 Hz), alpha (7.5–15 Hz), beta (15–30 Hz), lower gamma (30–60 Hz), and higher gamma (60–110 Hz) bands (*i.e.*, the highest band was less than one octave).

### Statistical analysis

RT and hit rate (HR) differences were analyzed using t-tests. The HR distributions were cubically and the RT distributions logarithmically transformed to render them approximately Gaussian [61], as confirmed by using the Jarque-Bera test. To test our specific hypotheses, we examined correlations between behavioral performance (RT to incongruent normalized by the RT to congruent trials) *vs*. cortical phase locking and power changes during periods preceding stimulus presentation and during the 500-ms period preceding the behavioral responses. For the pre-stimulus analyses, we calculated the correlations between the baseline preceding the incongruent trials and the normalized RT conflict effect. For the pre-response analyses, a 2×2 factorial design was utilized to control for the effects of responding hand (right *vs*. left) and task type (incongruent *vs*. congruent). To control for multiple comparisons, the resulting statistical estimates were tested against an empirical null distribution of maximum cluster size across 10,000 iterations with a vertex-wise threshold of *P*<0.05 and cluster-forming threshold of *P*<0.05 (Bonferroni corrected by the number of hemispheres), yielding clusters corrected for multiple comparisons across the surface.

## Results

The behavioral analyses showed a significant conflict effect, demonstrating that the task was functioning as anticipated. RTs to the incongruent trials were significantly longer than those to the congruent trials (*t*_9_ = 7.5, *p*<0.001; mean ± SD *RT*_Congruent_ = 569±116 ms; mean ± SD *RT*_Incongruent_ = 637±118 ms), and HRs were significantly lower to the incongruent than congruent trials (*t*_9_ = −3.2, *p* = 0.01; mean ± SD *HR*_Congruent_ = 98±2%; mean ± SD *HR*_Incongruent_ = 95±4%).

### Seed-based phase locking patterns correlating with behavioral conflict processing

**Figure 2:**
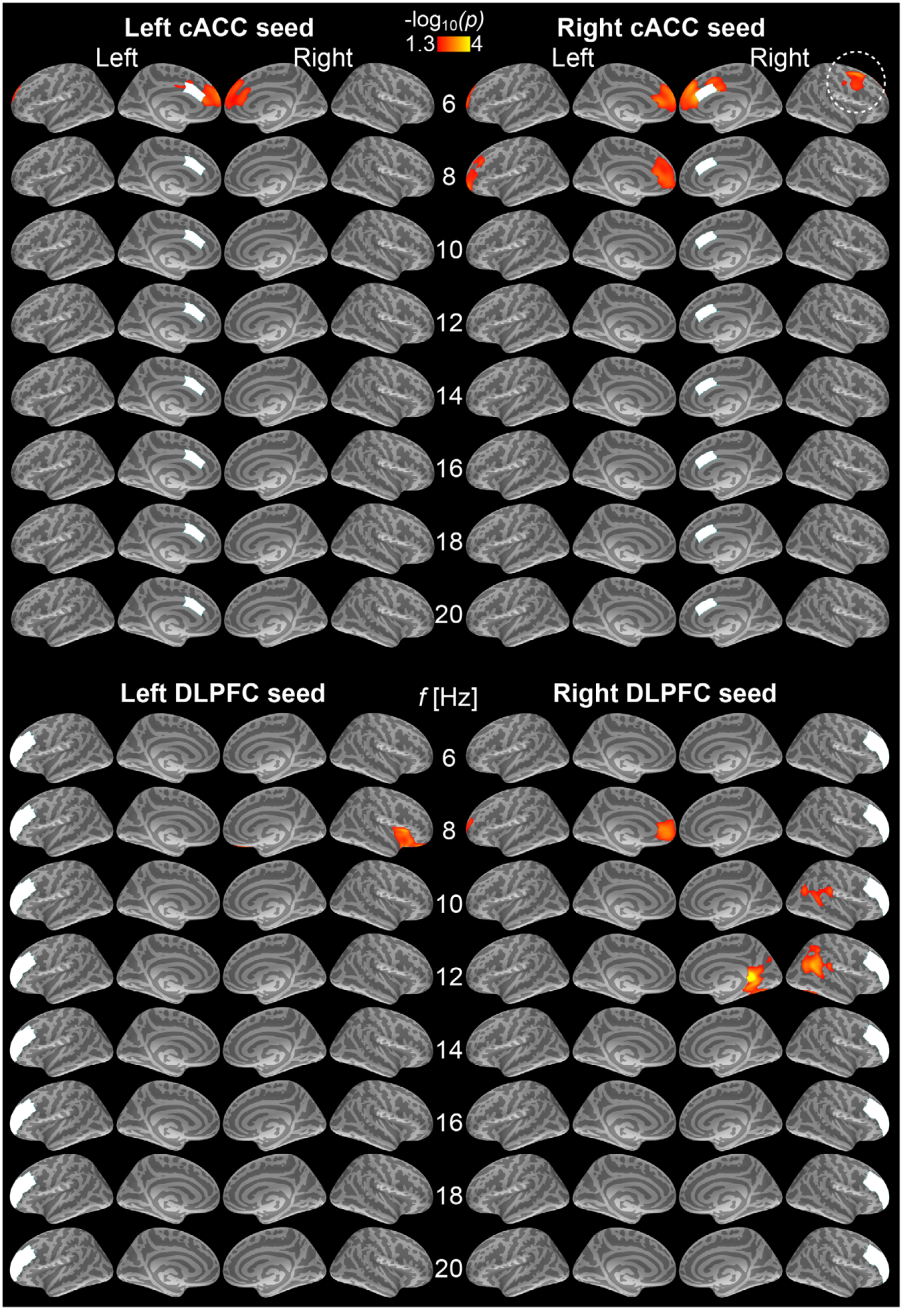
Baseline correlations between conflict-induced RT lags and cortical phase locking to the caudal anterior cingulate (cACC) and dorsolateral prefrontal cortex (DPLPC) seeds during 500-ms periods preceding conflicting trials. All significant findings reflect correlations between reduced RT lags and increased seed-based phase locking patterns (*p*<0.05, cluster-based Monte Carlo simulation test). (Top) Left and right cACC seed data. Interestingly, at 6 Hz (i.e., theta), faster RTs after conflicting sounds correlated with increased connectivity between the right cACC and right lateral prefrontal areas (encircled), and between bihemispheric cACC seeds and adjacent medial frontal cortex locations. (Bottom) Left and right DLPFC seed data. Faster RTs after incongruent trials were strongly correlated with increased alpha phase locking between the right DLPFC and right retrosplenial (12 Hz) and inferior parietal/TPJ regions (10–12 Hz), as well as at 8 Hz, between the left DLPFC and right anterior insula and between right DLPFC and left anteromedial frontal cortex. The results reflect −log_10_(*p*) of the initial *t*-statistics, masked to the locations where the cluster-based Monte Carlo simulation test was significant (*p*<0.05).

Our main purpose was to examine cortico-cortical functional connectivity patterns that predict efficient behavioral performance during the modified auditory flanker task. This analysis concentrated on two distinct time periods, preceding the stimulus trials and preceding each behavioral response that followed the stimuli. **Figure 2** shows estimates of “sustained” baseline connectivity patterns, which predicted fast and efficient conflict processing, i.e., reduced RT lags, in the subsequent stimulus trials. Significant correlations (*p*<0.05, cluster-based Monte Carlo simulation test) between reduced RT lags and increased phase locking patterns (debiased wPLI) were observed at the theta range (6 Hz) between the right cACC and right lateral frontal regions, including FEF. Reduced conflict-induced RT lags also correlated with increased theta/alpha connectivity between cACC and medial frontal cortex regions bilaterally at 6 Hz, and between the right cACC and left medial/superior frontal cortex at 8 Hz. Note that in this case the connectivity patterns are unlikely to be explained by biases caused by the anatomical adjacency, i.e., EEG/MEG point spread and cross talk, because the debiased wPLI index emphasizes synchronization patterns with nonzero phase lags [58].

During the pre-stimulus baseline period, the strongest behavioral correlations involving the DLPFC seed connectivity patterns were observed at the alpha range (10–12 Hz): Reduced RT lags associated with incongruent trials correlated with increased phase locking between the right DLPFC and right inferior parietal/temporoparietal junction (TPJ), as well as the right retrosplenial cortices. In addition, analogous behavioral correlations were observed with increased 8-Hz phase locking between the left DLPFC and right anterior insula as well as with phase locking between the right DLPFC and left anterior medial frontal cortex. **Table 1** shows the details of the cluster statistics reflecting correlations between the conflict-induced RT lags and the phase locking differences after incongruent vs. congruent stimuli, as measured from the 500-ms periods preceding each stimulus.

**Table 1:** Correlations between RT lags caused by conflict and seed-based phase locking patterns during the pre-stimulus baseline period.

Target hemisphere	Seed	f [Hz]	Max value	Size (mm^2^)	*x*_MNI_	*y*_MNI_	*z*_MNI_	Cluster-wise *P*	Anatomical coverage
**Left**									
	Left cACC								
		6	−2.2	3922	−8	58	17	0.0022	Superior frontal
	Right cACC								
		6	−2.4	4118	−5	38	8	0.0017	Medial frontal
		8	−2.2	6661	−6	35	8	0.0001	Medial frontal
	Right DLPFC								
		8	−2.4	3347	−7	42	2	0.0116	Medial frontal
**Right**									
	Left cACC								
		6	−2.0	3149	9	41	35	0.0111	Superior frontal
	Right cACC								
		6	−3.0	4605	6	33	18	0.0001	Medial frontal
		6	−2.3	3126	27	8	47	0.0112	Lateral prefrontal
	Left DLPFC								
		8	−2.5	3657	26	12	−17	0.0047	Anterior insula, IFC, orbitofrontal
	Right DLPFC								
		10	−1.9	2930	47	−35	28	0.0155	TPJ/inferior parietal
		12	−3.7	4975	14	−48	3	0.0001	Retrosplenial/precuneus
		12	−2.8	5534	44	−54	23	0.0001	IPS, TPJ/inferior parietal

Monte Carlo simulation results of the data in **Fig. 2** are demonstrated. The maximum values of initial GLM reflect −log10(p)×sign(*t*).

**Figure 3:**
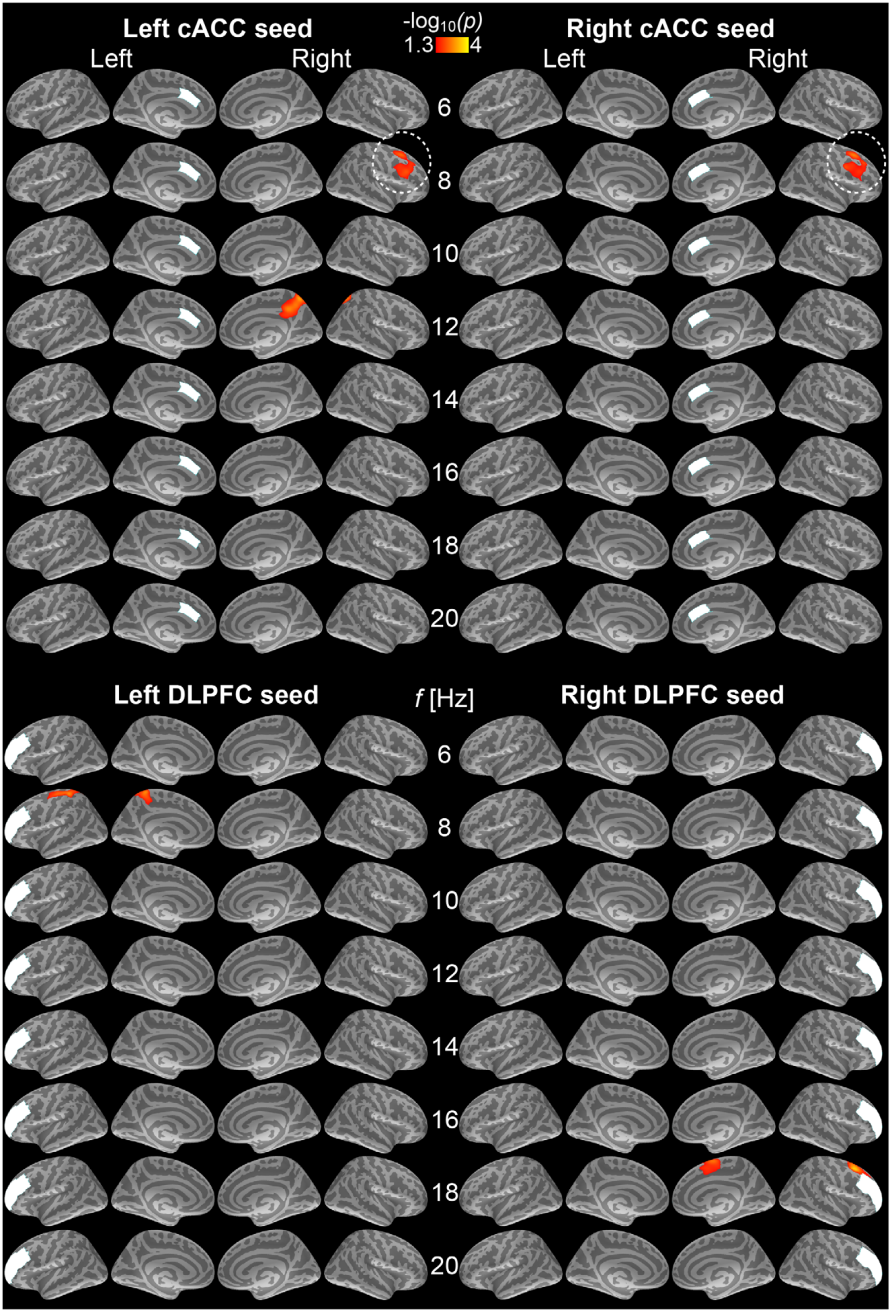
Correlations between conflict-induced RT lags and phase locking to the caudal anterior cingulate (cACC) and dorsolateral prefrontal cortex (DPLPC) seeds during 500-ms periods preceding incongruent trials. All statistically significant findings reflect correlations between reduced RT lags and increased seed-based phase locking after incongruent vs. congruent trials (*p*<0.05, cluster-based Monte Carlo simulation test). (Top) Left and right cACC seed data. Most interestingly, at 8 Hz, reduced conflict-induced RT lags correlated with increased connectivity between the bilateral cACC and right lateral prefrontal areas encompassing FEF and posterior aspects of DLPFC (encircled). At 12 Hz, reduced conflict-induced RT lags also correlate with increased phase locking between the left cACC and right medial parietal cortex. (Bottom) Left and right DLPFC seed data. Reduced conflict-induced RT lags correlated with increased 8-Hz alpha phase locking between the left DLPFC and left postcentral/precentral regions, as well as increased 18-Hz beta phase locking between the right DLPFC and right superior frontal/pre-supplementary motor area (pre-SMA) regions. The results reflect −log_10_(*p*) of the initlal *t*-statistics, masked to the locations where the cluster-based Monte Carlo simulation test was significant (*p*<0.05).

**Figure 3** shows the results reflecting efficient conflict processing, during the post-stimulus/pre-response period, as estimated from a full factorial model estimating the correlations between the RT lags to incongruent vs. congruent trials and phase locking differences after incongruent vs. congruent stimuli. Most importantly, consistent with the hypothesis that efficient conflict processing depends on cingulofrontal connectivity, we found that reduced conflict-induced RT lags correlated with increased 8-Hz alpha phase locking between the bilateral cACC seeds and right lateral prefrontal regions. In the right lateral prefrontal cortex, these behavioral correlation patterns encompassed the likely location of right FEF, associated with the dorsal attention network, and the right DLPFC. In addition, behavioral correlations emerged with increased phase locking between the following regions: the left cACC and right medial parietal cortex at 12 Hz, left DLPFC and left superior/medial pre- and post-central regions at 8 Hz, and right DLPFC seed and right superior frontal gyrus regions at 18 Hz.

**Figure 4:**
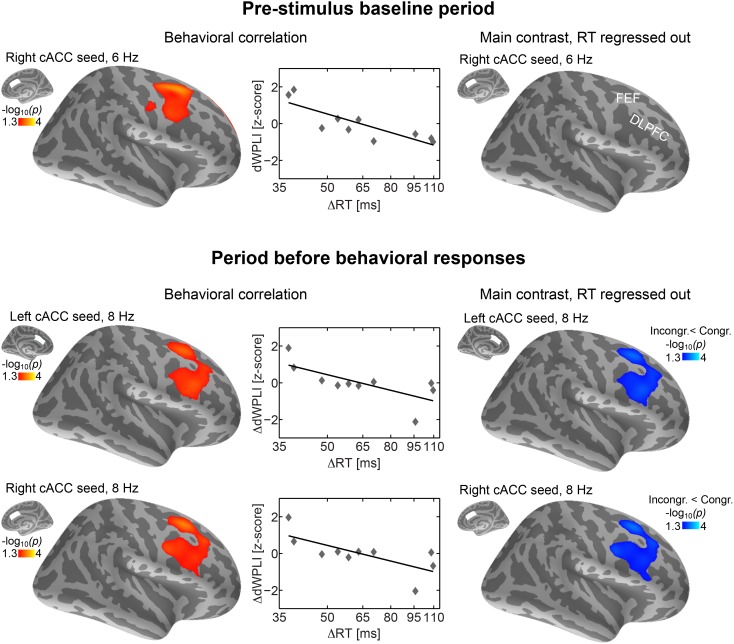
Behavioral conflict resolution and theta/alpha connectivity patters between medial and lateral frontal cortices. (**Top**) The results during the baseline periods. The connectivity between cACC and lateral frontal cortex, specifically FEF, at 6 Hz predicted faster conflict processing, possibly associated with sustained allocation of attention that is necessary for efficient conflict monitoring and resolution during selective listening. The scatter diagram shows the ΔRT (natural logarithmic scale) plotted against lateral frontal cluster average debiased weighted phase lag index (wPLI), with a best-fitting linear trend, depicting the correlation between fast conflict resolution and cACC-lateral frontal theta phase locking. (**Bottom**) Analyses during the 500-ms period preceding each behavioral response are shown. Stronger connectivity between the bilateral cACC and right lateral frontal cortices (FEF, DLPFC) at 8 Hz predicted fast conflict resolution performance, i.e., reduced ΔRT between incongruent and congruent trials. When the effect of behavioral variability was covaried out, the main contrast showed a significant decrease of post-conflict connectivity between the approximately same regions. The scatter diagrams show the ΔRT (natural logarithmic scale) plotted against the lateral frontal debiased wPLI cluster averages for the cACC seed, with the best-fitting linear trend.

**Figure 4** highlights the effects, concentrating on the theta/alpha phase locking between the cACC seeds and right lateral frontal cortices before (at 6 Hz) and after the occurrence of the conflicting information (at 8 Hz), which predict efficient/fast conflict processing performance. In addition to the significant behavioral correlations, the data in **Figure 4** also show that when the contribution of behavioral performance has been removed from the model, during the post-stimulus/pre-response period of the main contrast, a significant (*p*<0.05, cluster-based Monte Carlo simulation test) decrease of debiased wPLI between the cACC and right lateral frontal regions is observed after the incongruent vs. congruent trials. This leaves room for an interpretation that the occurrence of a conflicting information has a tendency to disrupt an existing phase locking pattern between the cACC and lateral frontal cortex, rather than eliciting a new functional connectivity pattern, and that the resolution of a conflict depends on the restoration of the sustained functional coupling between the medial and lateral frontal regions. **Table 2** shows the cluster statistics of correlations between conflict-induced RT lags and connectivity patterns emerging during the 500-ms periods preceding each behavioral response.

**Table 2:** Correlations between the RT lag caused by conflict and seed-based phase locking differences between periods preceding behavioral responses to incongruent vs. congruent sounds.

Target hemisphere	Seed	f [Hz]	Max value	Size (mm^2^)	*x*_MNI_	*y*_MNI_	*z*_MNI_	Cluster-wise *P*	Anatomical coverage
**Left**									
	Left DLPFC								
		8	−2.4	3659	−28	−35	58	0.0038	SMA, postcentral
**Right**									
	Left cACC								
		8	−1.9	3325	29	13	46	0.0095	Lateral PFC, FEF
		12	−2.6	2983	5	−63	55	0.0130	Precuneus
	Right cACC								
		8	−2.1	3740	29	13	46	0.0018	Lateral PFC, FEF
	Right DLPFC								
		18	−2.7	3531	28	14	44	0.0068	Lateral PFC, FEF

Monte Carlo simulation results of the data in **Fig. 3** are demonstrated. The maximum values of initial GLM reflect −log10(p)×sign(*t*).

### Cortical power mapping patterns correlating with behavioral conflict resolution

**Figure 5:**
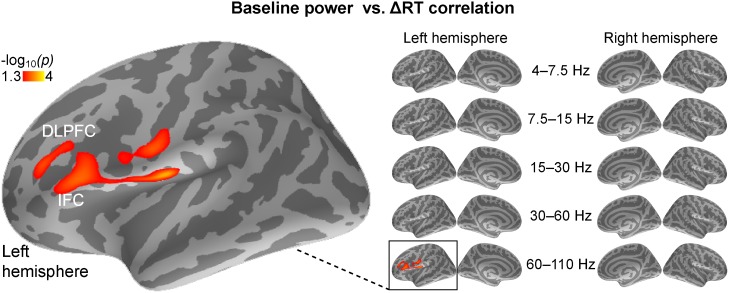
Correlation analyses between the power of pre-stimulus baseline oscillations and behavioral performance. Efficient behavioral conflict processing, as reflected by shorter conflict-induced RT lags, correlated with increased oscillatory power at the higher (60–110 Hz) gamma band during the pre-stimulus period (*p*<0.05, cluster-based Monte Carlo simulation test). The significant correlation were concentrated in the left lateral and inferior frontal cortices, consistent with previous fMRI studies suggesting their role in speech-sound related auditory attention tasks [102]. No significant correlations were observed in the other frequency bands, in the other brain regions, or during the periods immediately before the behavioral responses. The figure shows the significance of initial GLM, masked to the locations that survived the post-hoc correction based on the cluster-based Monte Carlo simulation test.

Finally, we also correlated behavioral measures with cortical power mapping results during the same time periods that were examined in the functional connectivity analyses. These analyses revealed a significant correlation between increased left lateral prefrontal cortex high-gamma power (60–110 Hz) during the pre-stimulus baseline period and reduced conflict-induced RT lags (**Figure 5**). No significant correlations emerged in the other frequency bands during the baseline period, or in any frequency band during the period preceding the behavioral responses.

## Discussion

We studied seed-based oscillatory phase locking patterns associated with conflict processing during an auditory flanker interference task. Consistent with our hypothesis, the results demonstrate that efficient conflict resolution during sound object selection, as measured with reduced conflict-induced RT lags, is predicted by theta/alpha phase coupling between the cACC and right lateral frontal cortex regions intersecting FEF and DLPFC. Notably, the correlation patterns observed were not fully consistent with a hypothesis that cACC is a region only transiently recruited to detect conflicting coactivations of information processing streams. On the contrary, our statistical model showed that, when the variability of behavioral performance was regressed out, the occurrence of conflict actually disrupts the sustained connectivity between the cACC and lateral frontal regions. Our results, thus, seem to suggest that the cACC is involved in networks that bias processing of task-relevant sound information in situations requiring sustained engagement of control mechanisms.

It is generally agreed that cognitive control utilizes a network of brain regions including DLPFC, ACC, and posterior parietal regions [62]. Further, an emerging view is that discrete areas process information in a coordinated manner rather than as distinct modules [63]. Coordinated activity between neurons in different networks and brain regions may be mediated by spike synchrony with local field potential (LFP) oscillations [64] as well as synchrony of LFP oscillations, which provide windows of time when the effectiveness of a proximal spike on a distal neuron's postsynaptic potential is enhanced [20], [63], [65]. Our finding of the cACC phase-locked with the FEF and portions of DLPFC in theta/alpha band during conflict resolution is in line with this proposal, and further demonstrates a functional correlation of cognitive control processes and oscillatory theta-band activity between the ACC, the FEF, and the DLPFC.

Our results that the ability to allocate attention to relevant sound objects and disregard those with conflicting information correlates with cACC connectivity with lateral frontal cortices are consistent with previous studies that link cACC with conflict processing [66]–[68], error-related reinforcement learning [69], selection of goal-directed actions [70], and allocation of attentional resources [71], [72]. Although most previous studies consider vision, there is an abundance of evidence that ACC also plays a role in auditory attention and cognitive control [12], [31]–[33]. For example, there is evidence [73] that conflict-related EEG activations, which presumably originate in ACC [74], correlate with the degree of ambiguity of choice-tasks trials that do not involve actual conflicts. This suggests that the underlying circuits might also contribute to disambiguation of complex stimulus scenes. Further, conflict-related mid frontal theta oscillations have been previously identified in a number of EEG studies [27], [75]. By exploiting the temporal and spatial resolution of combined MEG/EEG methods and anatomical MRI, our findings provide convincing evidence regarding the source of the mid frontal theta, as well as its involvement in early stage of perceptual processing before the response selection. Importantly, our findings extend previous literature and show that the functional network involving cACC, FEF, and DLPFC, not only monitors instantaneous occurrences of conflicts, but also exerts sustained attentional control needed for the selection of relevant sound objects in complex auditory environments.

During the post-stimulus/pre-response period, most significant correlations with cACC theta/alpha phase locking patterns emerged in the right FEF. Accumulating evidence suggests that the right FEF is a key part of the dorsal attention network (DAN) that governs voluntary deployment of spatial attention to task-relevant locations (for a review, see [76]), and this area could also be associated with auditory spatial attention [77]. Recent neurophysiological studies in non-human primates further suggest that FEF initiates top-down signals that helps bias saliency maps in posterior parietal cortices (e.g., in the monkey homologue of human intraparietal sulcus, IPS) that prioritize stimuli occurring in task relevant locations [78]. One might therefore conclude that cACC provides top-down feedback that, analogously to its proposed role during visual attention [79], helps the network involving FEF and IPS boost attention toward sound objects originating from the task-relevant spatial direction (here, straight ahead) and suppress those from task-irrelevant directions (here, 45° right/left) during auditory attention. Notably, it has been long speculated that altered connectivity of cingulate regions with FEF and parietal cortices underlies impaired selective attention in schizophrenia, reflected by the patients’ inability to distinguish relevant vs. irrelevant objects in their perceptual field [80].

In addition to FEF, we also found the cACC phase-locked with the posterior aspects of right DLPFC at the theta/alpha bands. This finding is consistent with the known bidirectional anatomical connections between ACC and DLPFC [81], and with observations that these two areas work in concert to enhance top-down control [82]. Our findings are also in line with a proposition that theta oscillations are the electrophysiological mechanism underlying these interactions [83]. For example, a recent animal study reported extensive interactions between ACC and lateral frontal cortices at the theta range [84]. An open question, however, is how the functions of ACC vs. DLPFC are orchestrated during attentional control in the face of interferences and conflicts. The “conflict monitoring” hypothesis [85] suggests a system where ACC monitors and detects conflicts caused by distracting stimuli, and signals them to DLPFC that exerts the ultimate control [86]. The present results are not fully consistent with this notion, as the significant correlation between efficient behavioral conflict resolution and cACC–DLPFC/FEF connectivity was also observable during the pre-stimulus period, that is, before the conflict had even occurred. The presence of effects during the baseline period resembles recent observations in monkey neurophysiological studies [87]. Moreover, when the between-subject behavioral variability was covaried out from our statistical models concerning the post-stimulus/pre-response period, it appeared that the occurrence of a conflict results in a transient decrease in cACC–DLPFC/FEF phase locking. Overall, the results would seem to be most consistent with the notion that cACC and DLPFC work together in a more sustained fashion to suppress distraction and boost attention to relevant stimuli.

However, it is important to note that the present MEG/EEG source estimates are likely less anatomically specific than those achievable with fMRI, not to mention invasive recordings. That is, there is fMRI evidence that the different subregions of ACC may be differentially recruited during conflict processing and distraction suppression. Specifically, a recent study [88] suggested that whereas dorsal aspects of ACC, which overlap with the present cACC seed regions, are associated with biasing attention toward relevant stimuli, the more rostral ACC areas were associated only with conflict detection.

Another interesting finding of the current study is the link between pre-stimulus alpha and performance. Specifically, during the pre-stimulus baseline period, it was found that increased alpha phase locking between the left DLPFC and right AI, as well as between the right DLPFC vs. left anterior MFC and right inferior parietal/TPJ, correlated with fast and efficient behavioral conflict. Although alpha power increases have been often linked to cortical “idling”, there is accumulating evidence that inter-regional phase locking at this frequency range mediates top-down effects of attention. Evidence supporting this notion has been shown in animal studies suggesting that interregional alpha and beta synchronization in areas roughly corresponding to primate DLPFC and ACC that generated top-down attentional signals [63], as well as in animal and human studies reporting increases of alpha coherence in visual cortices [29], [89] and strengthening of alpha phase synchrony between occipital visual cortex and parietal [90] or PFC [91] regions by attention.

At the beta frequency range, we found that auditory-behavioral conflict resolution correlated with increased phase locking between the right DLPFC and the right pre-SMA. The pre-SMA has anatomical connections with the DLPFC [92], [93], and these regions have been implicated in the internally generated aspects of action planning, such as choice and intention. Numerous studies have documented beta oscillations associated with movement behavior and response inhibition, but beta power has also been implicated in broader cognitive processes (for a review, see [94]). In fact, it has been proposed that inter-areal coherence, at the beta frequency range, may have an essential role in cognitive functions including selective attention, working memory, object recognition, perception or sensory-motor integration, due to the prominent role of beta-band oscillations in top-down projections [94]. Interestingly, there is evidence from modeling studies showing that synchronization can tolerate longer synaptic delays for beta than gamma oscillation [23], [95], suggesting that long-distance oscillatory synchronization may be more robustly realized at beta frequencies. Our data confirms and extends pervious findings of pre-SMA’s involvement in conflict situations [96] and demonstrates that synchronized beta-band oscillations may be the underlying neural mechanism supporting inter-regional communication during conflict processing. Future studies are needed to discern whether its exact role is related to resolving competition between motor plans during response selection, or to conflict-related information processing *per se*.

Our finding that the subjects’ ability to resolve conflicts between competing auditory inputs correlated with alpha-band phase locking between the right DLPFC and the retrospenial/precuneus cortex during the baseline period is consistent with recent resting state studies in monkeys and humans demonstrating functional connectivity between the DLPFC and the retrospenial/precuneus regions [97]. It has been proposed that the retrospenial cortex might be the neural substrate between the interaction between the DLPFC and hippocampus [98], and that it plays an essential role in cognitive context processing [for a review, see 99]. For example, in an fMRI/MEG study investigating neural synchronization in the contextual processing, activations associated with stronger contextual association were found in retrospenial, parahippocampal and occipital cortices. Importantly, greater phase locking at beta band between these regions were also observed. Taken together, we speculate that the increased phase locking between the right DLPFC and the retrospenial/precuneus cortex observed in the current study might reflect enhanced contextual processing originates in the DLPFC in the face of increasing conflicts/interferences.

Notably, there was a significant correlation between efficient behavioral conflict resolution and increased oscillatory power at the higher (60–110 Hz) gamma band, which concentrated in the left inferior frontal cortex (IFC) during the pre-stimulus period. Previous fMRI studies have shown that IFC are associated with proactive interference [100], [101] and may play an important role in speech-related auditory attention tasks [102]. Further, several neurophysiological studies have also observed sustained gamma oscillations in left IFC associated with phonological processing [103] and maintaining auditory patterns in short-term memory [104]. Our results suggest that IFC gamma oscillations may be essential in maintaining relevant auditory objects during selective attention tasks, and support the concept that attention can rely on gamma-band synchronization that helps integrate neural activities related to a specific sensory object into a stable, salient and coherent representation [19]. More generally, this result is consistent with previous findings associating sustained prefrontal gamma-power increases with successful auditory attention performance [38].

Finally, it is noteworthy that the degree of conflict in each individual trial is essentially modulated by what happened during the preceding trials. The conflict monitoring theories [105] suggest that such a “conflict adaptation’ occurs because the preceding conflicts trigger stronger top-down control, which improves performance on subsequent trials of similar context. An alternative theory, however, suggested that these effects are related to a priming effect through episodic memory [106]. Although the exact mechanisms may still be debatable, the trial structure is nevertheless a factor that could affect responses and brain activations at the level of individual trials. Relevantly to the current study, previous fMRI studies have shown that the DLPFC activity gets stronger with an increasing predictability and decreasing experience of conflict, that is, when the probability of conflicts increases [9] or an increasing number of incongruent trials occur in a row during visual flanker task studies, respectively [11], [107], [108]. Future studies with runs having different probabilities of conflict could help elucidate how such a factor may affect cortico-cortical phase locking between cACC and DLPFC.

## Conclusions

Our results demonstrate the importance of sustained connectivity, observed as theta/alpha phase locking between ACC and lateral frontal cortices including the right FEF and DLPFC, for efficient auditory conflict processing, and suggest brain regions of control and attention networks may communicate through synchronous oscillations to manage auditory scenes with multiple overlapping signals. The broader functional relevance of this observation is supported by recent results that pre-stimulus preparatory cACC–FEF alpha coherence is reduced in subjects with autism spectrum disorders during antisaccade tasks [109]. In the context of previous theories, the present results are most consistent with a prediction that ACC may play a role in “regulatory” networks that suppress distractions and boost attention to relevant objects in multisource acoustic environments. The cortical power estimates suggest that gamma-band activity in the left IFC plays a role in selective attention to speech-sound objects.
